# Supplementary material for: Tamarind Xyloglucan Oligosaccharides Attenuate Metabolic Disorders via the Gut–Liver Axis in Mice with High-Fat-Diet-Induced Obesity
Source: Foods. 2023 Mar 24;12(7):1382. doi: 10.3390/foods12071382 (PMC10093524; doi:10.3390/foods12071382)
Supplement: Supplementary file 1 [file foods-12-01382-s001.zip › foods-2203258-supplementary.pdf]

Supplementary data

Tamarind Xyloglucan Oligosaccharides Attenuate Metabolic Disorders via the Gut–Liver Axis in Mice with High-Fat-Diet-Induced Obesity

Chun-Hua Zhu [1], Yan-Xiao Li [2,3], Yun-Cong Xu [1], Nan-Nan Wang [1], Qiao-Juan Yan [2] and Zheng-Qiang Jiang [1,*]

[1] Department of Nutrition and Health, College of Food Science and Nutritional Engineering, China Agricultural University, Beijing 100083, China
[2] Key Laboratory of Food Bioengineering (China National Light Industry), College of Engineering, China Agricultural University, Beijing 100083, China;
[3] College of Food Science and Engineering, Collaborative Innovation Center for Modern Grain Circulation and Safety, Nanjing University of Finance and Economics, Nanjing 210023, China
* Correspondence: zhqjiang@cau.edu.cn; Fax: +86-10-82388508

Supplementary Table S1. Diet and gavage dose of treatment in different groups.

Groups	Diet and intervention dose
Normal diet group (ND)	10 kcal% fat diet+ same volume of pure water
High-fat diet group (HFD)	60 kcal% fat diet + same volume of pure water
High-fat diet group + orlistat (HFDA)	60 kcal% fat diet + orlistat (recommended dose)
High-fat diet group + high dose of TXOS (HFDH)	60 kcal% fat diet + 4.8 g/kg of TXOS
High-fat diet group + middle dose of TXOS (HFDM)	60 kcal% fat diet + 3.2 g/kg of TXOS
High-fat diet group + low dose of TXOS (HFDL)	60 kcal% fat diet + 1.6 g/kg of TXOS

Supplementary Table S2. Diet composition of normal and high-fat diet.

Ingredient	Normal diet		High-fat diet(D12492)	
	g	Kal	g	Kal
Casein, 30 Mesh	200	800	200	800
L-Cystine	3	12	3	12
Corn Starch	550	2200	0	0
Maltodextrin 10	150	600	125	500
Sucrose	4	16	68.8	275.2
Cellulose, BW200	50	0	50	0
Soybean Oil	25	0	25	225
Lard	20	180	245	2205
Mineral Mix M1002	10	0	10	0
DiCalcium Phosphate	13	0	13	0
Calcium Carbonate	5.5	0	5.5	0
Potassium Citrate, 1 H_2O	16.5	0	16.5	0
Vitamin Mix V10001	1	4	10	40
Choline Bitartrate	2	0	2	0
FD&C Yello Dye#1	0.03	0		
FD&C Blue Dye#1			0.05	0
Total	1050.03	3812	773.85	4057

Supplementary Table S3. Primer sequences used for q RT-PCR.

Target gene	Primer sequences (5'-3')	Amplification size (bp)
GAPDH-F	CCTCGTCCCGTAGACAAAATG	133
GAPDH-R	TGAGGTCAATGAAGGGGTCGT	
PPARG-F	GCTCCAAGAATACCAAAGTGCG	218
PPARG-R	GCTTCAATCGGATGGTTCTTCG	
FASN-F	TGAATCAGCCCCACGCAGT	297
FASN-R	CCGAGTCAGTCTTGGAGGACAT	
SREBP1c-F	GACATGCTCCAGCTCATCAACA	245
SREBP1c-R	GACACGGACGGGTACATCTTTA	
ACACA-F	TTTGTTTGGTCGTGACTGCTCTG	226
ACACA-R	AGGATGTTCAACCTGTAGCCGAG	
LXR-F	CCACCATTGAGATCATGTTGCTA	208
LXR-R	ATGGCGATAAGCAAGGCATACT	
GPNMB-F	GCCAAGCGATTTCGTGATGT	151
GPNMB-R	AGTCCTTCCACCTGCCGTCT	
PPARA-F	CACTACGGAGTTCACGCATGT	166
PPARA-R	GTGACATCCCGACAGACAGGC	

F, forward primer; R, reverse primer; PPARG, peroxisome proliferator-activated receptor-γ; FASN, fatty acid synthase; SREBP1c, sterol-regulatory element-binding protein-1c; ACACA, acetyl-CoA carboxylases alpha; LXR, the liver X receptor; GPNMB, glycoprotein nonmetastatic melanoma protein B; PPARA, the nuclear receptor peroxisome proliferator-activated receptor α.

Supplementary Table S4. Effect of TXOS on the serum of HFD-induced mice.

Groups	TC (mmol/L)	TG (mmol/L)	HDL-C (mmol/L)	LDL-C (mmol/L)
N D	3.37 ± 0.83b	1.80 ± 0.31a	1.46 ± 0.20b	1.73 ± 0.46b
HFD	5.09 ± 0.76ab	2.01 ± 0.44a	1.61 ± 0.20ab	2.67 ± 0.49ab
HFDH	5.60 ± 1.23ab	1.86 ± 0.36a	1.83 ± 0.04ab	2.84 ± 0.78ab
HFDM	8.35 ± 2.22a	2.65 ± 0.20a	2.16 ± 0.23a	4.62 ± 1.37a
HFDL	7.29 ± 0.70ab	2.26 ± 0.34a	2.05 ± 0.13ab	4.10 ± 0.55ab
HFDA	6.67 ± 0.83ab	2.20 ± 0.56a	1.51 ± 0.31ab	3.76 ± 0.45ab

Note: Different lowercase letters represent significant differences (**P*-value < 0.05).

ND, normal diet group; HFD, High-fat diet group; HFDH, 60 kcal% fat diet + high dose of TXOS (4.8 g/kg); HFDM, 60 kcal% fat diet + high dose of TXOS (3.2 g/kg); HFDL, 60 kcal% fat diet + high dose of TXOS (1.6 g/kg); HFDA, 60 kcal% fat diet + orlistat.

Supplementary Table S5. Fatty acid compositions in HFD-fed mice after supplementation with TXOS (μg/g).

Types of fatty acid	ND	HFD	HFDA	HFDH	HFDL
Caprylic acid	0.57 ± 0.06aA	0.27 ± 0.06bB	0.24 ± 0.03bB	0.11 ± 0.02cC	0.19 ± 0.02bB
Decanoic acid	0.80 ± 0.05aA	0.83 ± 0.20aA	0.37 ± 0.17aA	0.92 ± 0.24aA	0.47 ± 0.15aA
Dodecanoic acid	2.78 ± 0.52aA	7.13 ± 2.43aA	1.26 ± 0.84bB	2.50 ± 0.62bB	2.55 ± 0.73bB
Tridecanoic acid	0.09 ± 0.03abAB	0.23 ± 0.10aA	0.08 ± 0.03bB	0.05 ± 0.01bB	0.07 ± 0.03bB
Tetradecanoic acid	84.76 ± 4.81aA	95.89 ± 23.32aA	59.16 ± 30.47abAB	24.63 ± 4.39bB	96.29 ± 1.78aA
Myristoleic acid	3.90 ± 0.21aA	3.07 ± 0.53abB	0.80 ± 0.46dE	2.13 ± 0.56bcC	1.74 ± 0.42cdD
Myristelaidic acid	1.42 ± 0.49bB	3.48 ± 0.50aA	0.92 ± 0.33bB	1.26 ± 0.75bB	0.96 ± 0.32bB
Pentadecanoic acid	9.62 ± 0.34aA	10.74 ± 2.95aA	4.98 ± 2.68aA	5.35 ± 1.74aA	8.06 ± 1.09aA
cis-10-Pentadecenoic acid	0.83 ± 0.12aA	0.93 ± 0.39aA	0.61 ± 0.51aA	0.46 ± 0.11aA	0.61 ± 0.05aA
trans-10-Pentadecenoic acid	1.46 ± 0.21aA	1.59 ± 0.79aA	1.05 ± 0.91aA	0.92 ± 0.26aA	1.03 ± 0.11aA
Hexadecanoic acid	777.27 ± 56.2aA	980.48 ± 485.33aA	590.94 ± 648.19aA	393.94 ± 126.71aA	943.87 ± 46.96aA
Palmitoleic acid	320.19 ± 7.28aA	370.70 ± 69.17aA	191.38 ± 157.38aA	255.39 ± 77.66aA	307.85 ± 52.53aA
Palmitelaidic acid	2.07 ± 0.16aA	4.44 ± 0.40aA	2.10 ± 1.31aA	2.37 ± 0.95aA	3.19 ± 0.29aA
Heptadecanoic acid	6.54 ± 0.57aA	12.80 ± 3.88aA	8.99 ± 4.83aA	8.33 ± 1.99aA	12.78 ± 1.06aA
cis-10-Heptadecenoic acid	22.96 ± 0.54aA	33.41 ± 7.48aA	19.16 ± 13.23aA	23.44 ± 9.29aA	33.29 ± 4.93aA
trans-10-Heptadecenoic acid	0.10 ± 0.01aA	0.10 ± 0.01aA	0.07 ± 0.01aA	0.07 ± 0.02aA	0.09 ± 0.01aA
Octadecanoic acid	294.00 ± 1.69aA	295.45 ± 17.02aA	324.97 ± 11.37aA	312.94 ± 24.08aA	348.64 ± 21.50aA
Oleic acid	1096.23 ± 2.87aA	1805.53 ± 366.03aA	1193.58 ± 682.18aA	1246.33 ± 268.68aA	1593.19 ± 266.54aA
cis-Vaccenic acid	960.99 ± 6.68aA	1562.08 ± 315.81aA	1051.95 ± 633.11aA	1083.37 ± 241.91aA	1371.18 ± 222.94aA
Elaidic acid	702.92 ± 1.30aA	1222.54 ± 271.39aA	804.85 ± 484.93aA	825.27 ± 177.95aA	1065.75 ± 174.14aA
trans-Vaccenic acid	715.37 ± 6.31aA	1238.63 ± 285.69aA	827.68 ± 498.59aA	814.72 ± 181.99aA	1098.28 ± 190.29aA
Linoleic acid	2746.43 ± 523.50aA	2338.35 ± 423.29aA	1242.27 ± 573.76bB	1251.02 ± 232.19bB	2220.51 ± 503.05aA
Linoelaidic acid	313.3 ± 29.98cC	467.54 ± 83.24aA	144.87 ± 23.39eE	191.24 ± 35.94dD	414.25 ± 45.82bB
trans-7-Nonadecenoic acid	1.79 ± 0.27aA	3.51 ± 1.42aA	2.09 ± 1.02aA	5.14 ± 1.90aA	2.64 ± 0.59aA
trans-10-Nonadecenoic acid	11.98 ± 0.79aA	9.37 ± 2.13aA	8.44 ± 4.43aA	8.40 ± 2.57aA	9.08 ± 1.86aA
Arachidic acid	89.55 ± 9.98aA	121.42 ± 45.69aA	91.97 ± 40.79aA	76.77 ± 40.98aA	87.00 ± 27.28aA
γ-Linolenic acid	57.25 ± 8.48aA	56.65 ± 10.75aA	31.97 ± 16.01aA	49.25 ± 29.01aA	69.83 ± 26.51aA

cis-11-Eicosenoic acid	64.77 ± 3.60aA	67.49 ± 15.19aA	81.35 ± 44.93aA	54.03 ± 15.14aA	60.99 ± 11.67aA
trans-11-Eicosenoic acid	10.76 ± 1.15aA	23.34 ± 7.00aA	18.20 ± 8.46aA	15.35 ± 5.14aA	16.48 ± 3.85aA
a-Linolenic acid	158.76 ± 18.38aA	174.49 ± 21.07bB	64.31 ± 41.74cC	82.14 ± 21.46cC	133.07 ± 30.38bB
Heneicosanoic acid	17.72 ± 1.57aA	8.33 ± 3.13aA	12.25 ± 6.50aA	12.08 ± 1.41aA	14.91 ± 1.44aA
cis-11,14-Eicosadienoic acid	19.85 ± 1.56aA	26.01 ± 4.23aA	35.80 ± 18.54aA	26.89 ± 5.79aA	26.88 ± 3.94aA
Docosanoic acid	14.79 ± 0.04aA	15.26 ± 4.69aA	15.49 ± 2.10aA	13.63 ± 3.01aA	14.22 ± 2.52aA
Brassidic acid	46.85 ± 3.42aA	37.50 ± 12.42aA	34.69 ± 13.48aA	34.52 ± 16.04aA	30.00 ± 10.20aA
cis-11,14,17-Eicosatrienoic acid	60.13 ± 6.55aA	63.13 ± 13.38aA	85.05 ± 47.24aA	56.07 ± 15.03aA	70.19 ± 5.47aA
Arachidonic acid	555.48 ± 11.28aA	514.25 ± 82.18aA	564.09 ± 225.61aA	521.85 ± 90.94aA	779.10 ± 36.93aA
Tricosanoic acid	0.30± 0.01aA	0.40 ± 0.04aA	0.31 ± 0.08aA	0.34 ± 0.05aA	0.33 ± 0.01aA
cis-13,16-Docosadienoic acid	12.68 ± 1.09aA	10.42 ± 3.66aA	12.66 ± 5.07aA	12.10 ± 5.22aA	9.05 ± 2.65aA
cis-5,8,11,14,17-Eicosapentaenoic acid	74.62 ± 8.33cC	141.24 ± 14.56aA	34.50 ± 25.64eE	46.00 ± 18.64dD	93.31 ± 15.36bB
cis-7,10,13,16-Docosic acidtraenoic acid	37.76 ± 0.42aA	49.96 ± 5.35aA	64.13 ± 37.53aA	51.22 ± 12.52aA	49.64 ± 5.64aA
cis-7,10,13,16,19-Docosapentaenoic acid	54.62 ± 0.39aA	78.47 ± 8.61aA	47.44 ± 29.97aA	44.21 ± 15.39aA	70.33 ± 11.84aA
cis-4,7,10,13,16-Docosapentaenoic acid	28.87 ± 1.29aA	34.52 ± 7.44aA	55.07 ± 26.95aA	60.36 ± 32.17aA	42.66 ± 11.34aA
Tetracosanoic acid	0.28 ± 0.03bB	0.34 ± 0.03aA	0.16 ± 0.01cC	0.22 ± 0.06bB	0.28 ± 0.05bB
Nervonic acid	20.18 ± 0.48aA	7.55 ± 3.26cC	11.05 ± 1.63bB	13.79 ± 5.02bB	9.58 ± 0.54cC
cis-4,7,10,13,16,19-Docosahexaenoic acid	490.62 ± 28.38aA	491.25 ± 58.55aA	380.19 ± 91.04aA	327.53 ± 65.32aA	527.48 ± 126.23aA
homo-γ-Linolenic acid	97.33 ± 3.97aA	92.09 ± 22.13aA	95.55 ± 38.73aA	89.06 ± 33.38aA	84.85 ± 11.76aA
Petroselinic acid	0.91 ± 0.10aA	2.22 ± 0.27aA	2.14 ± 1.12aA	1.42 ± 0.45aA	2.33 ± 0.31aA

Data expressed as mean ± standard deviation (n=3).

Different lowercase letters in the same row indicate significant differences (**P*-value < 0.05).

Different uppercase letters in the same row indicate extremely significant levels (***P*-value < 0.01).

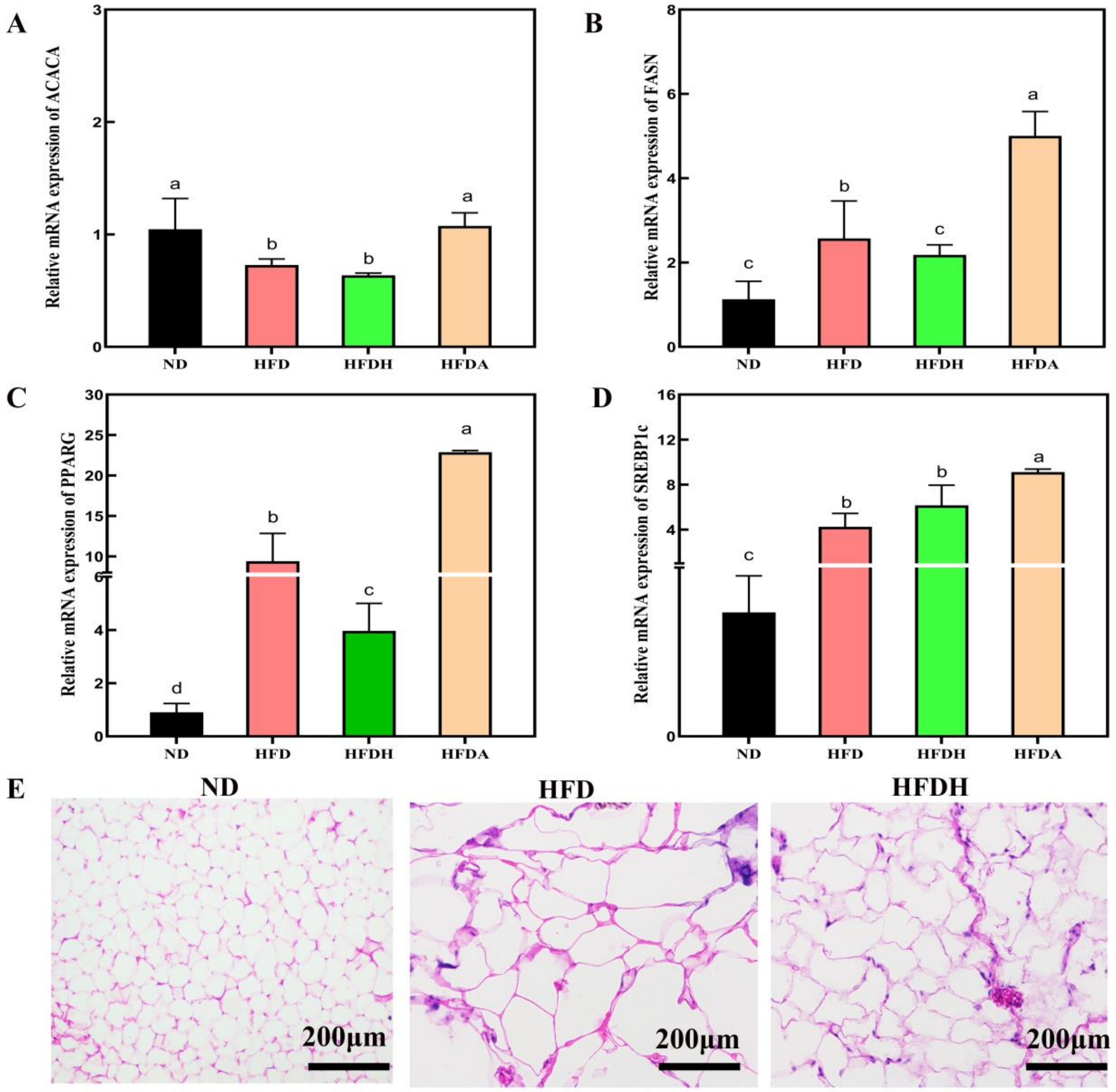

Supplementary Figure S1: Regulation of TXOS on adipose tissue and lipid metabolism-related gene expression in HFD-fed mice. (A) Relative mRNA expression of ACACA; (B) Relative mRNA expression of FASN; (C) Relative mRNA expression of PPARG; (D) Relative mRNA expression of SREBP1c; (E) Hematoxylin-eosin staining of epididymal adipose tissue (200×).

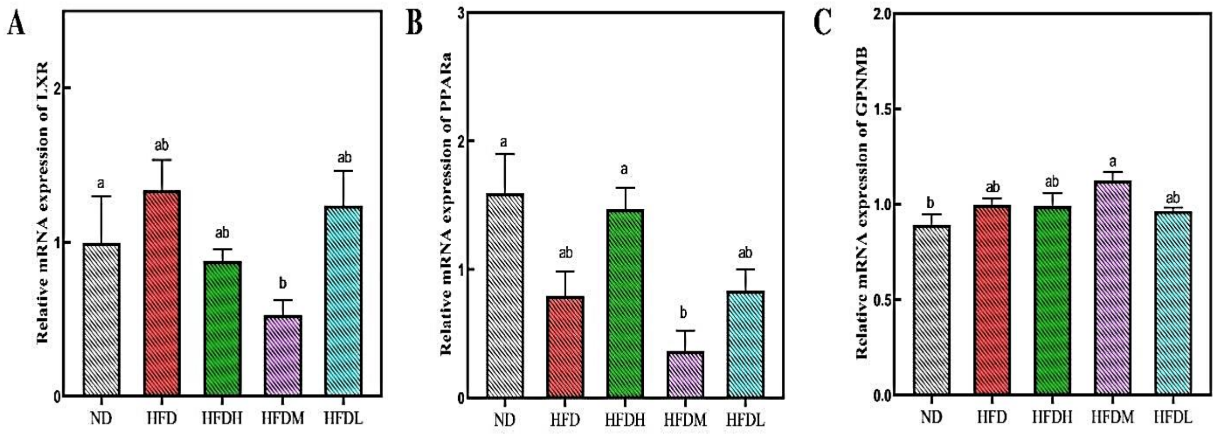

Supplementary Figure S2: Effect of TXOS on liver tissue and lipid metabolism-related gene expressions in HFD-fed mice. (A) Relative mRNA expression of LXR; (B) Relative mRNA expression of PPAR a; (C) Relative mRNA expression of GPNMB.

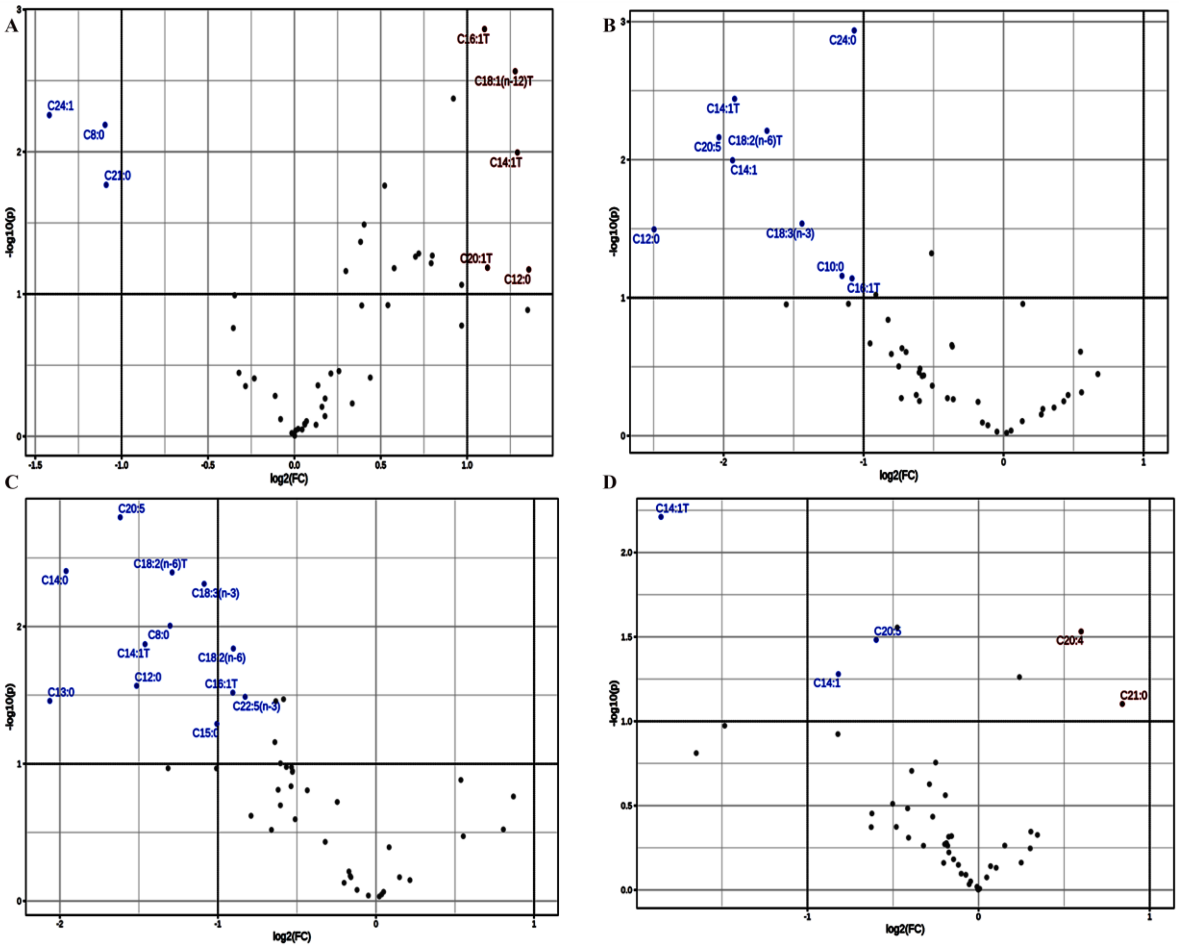

Supplementary Figure S3: Effect of TXOS supplementation on liver fatty acids among different mice groups. (A)Volcano plot showing the changes of liver fatty acids in HFD vs ND (fold change ≥ 2.0); (B) Volcano plot showing the changes of liver fatty acids in HFDA vs HFD (fold change ≥ 2.0); (C) Volcano plot showing the changes of liver fatty acids in HFDH vs HFD (fold change ≥ 2.0); (D) Volcano plot showing the changes of liver fatty acids in HFDL vs HFD (fold change ≥ 2.0).

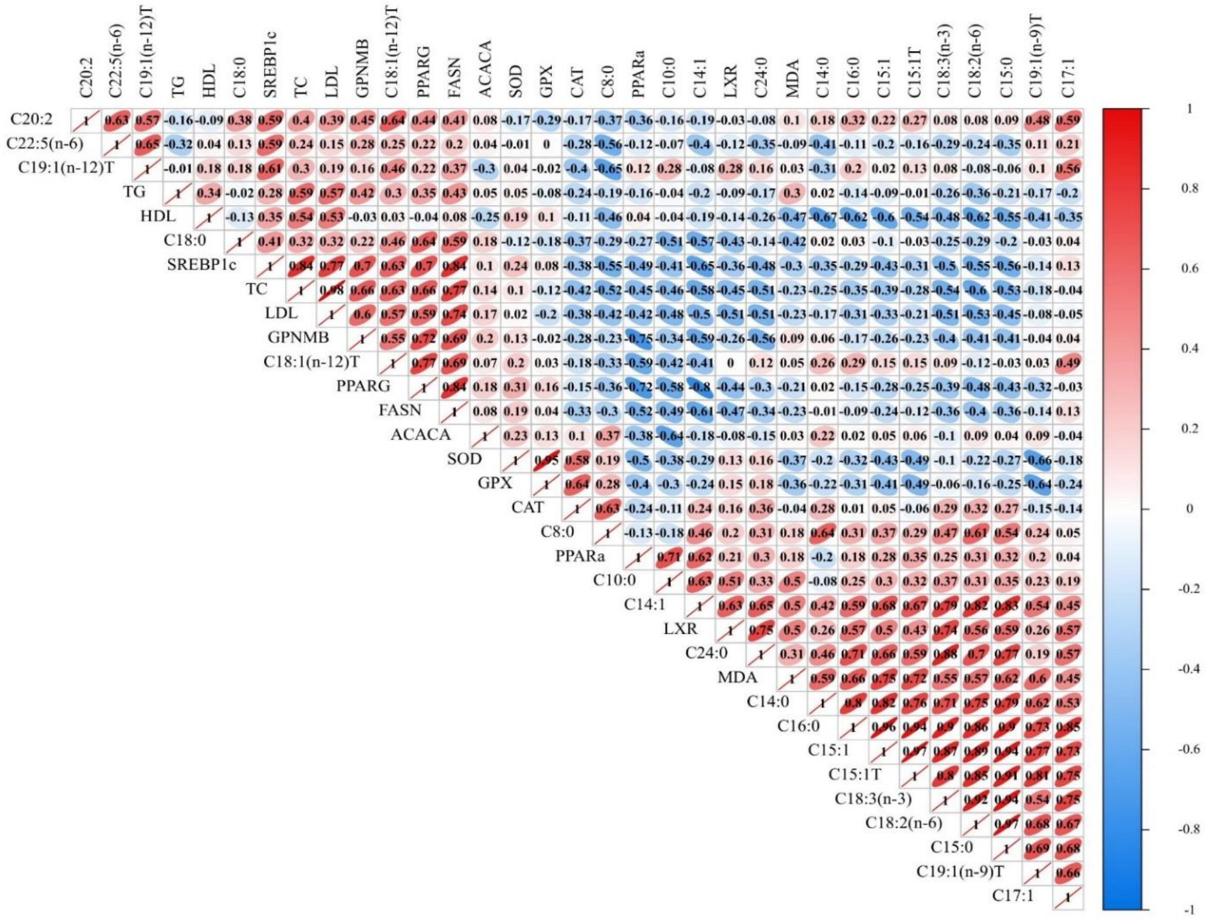

Supplementary Figure S4: Spearman’s correlation analysis between the obesity parameters and the significantly altered fatty acids in different groups.

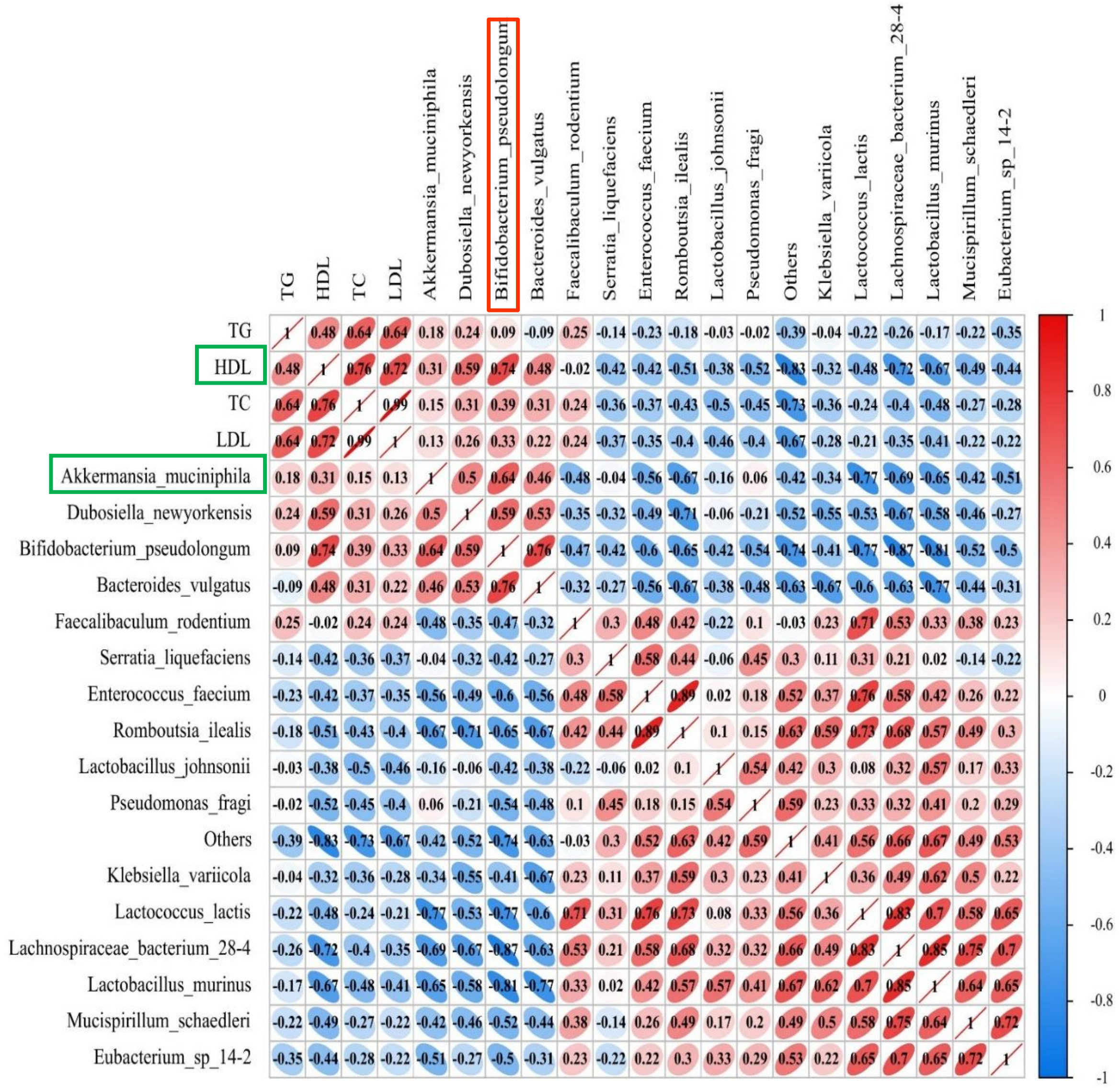

Supplementary Figure S5: Spearman's correlation analysis between species of gut bacteria and serum biochemical parameters.

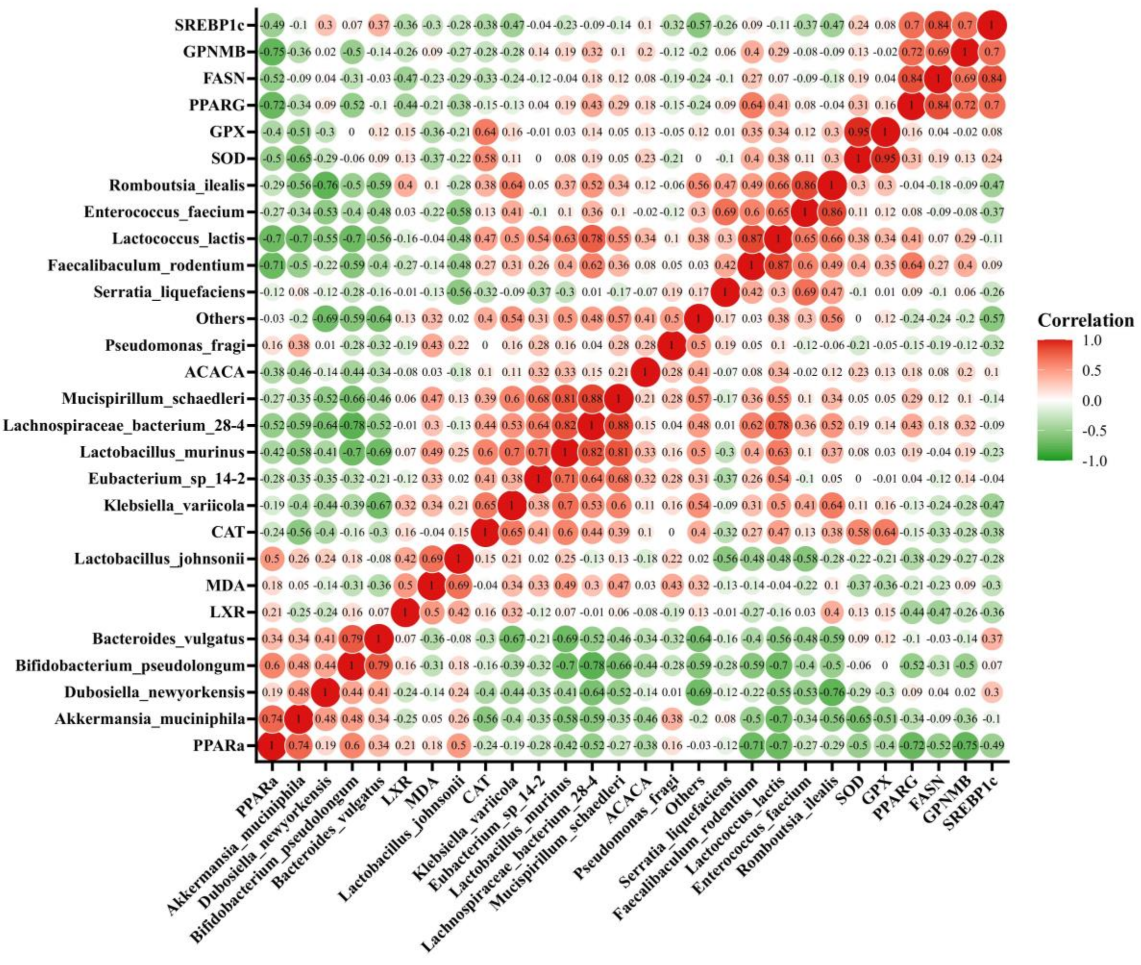

Supplementary Figure S6: Spearman's correlation analysis between species of gut bacteria and genes in liver and adipose.

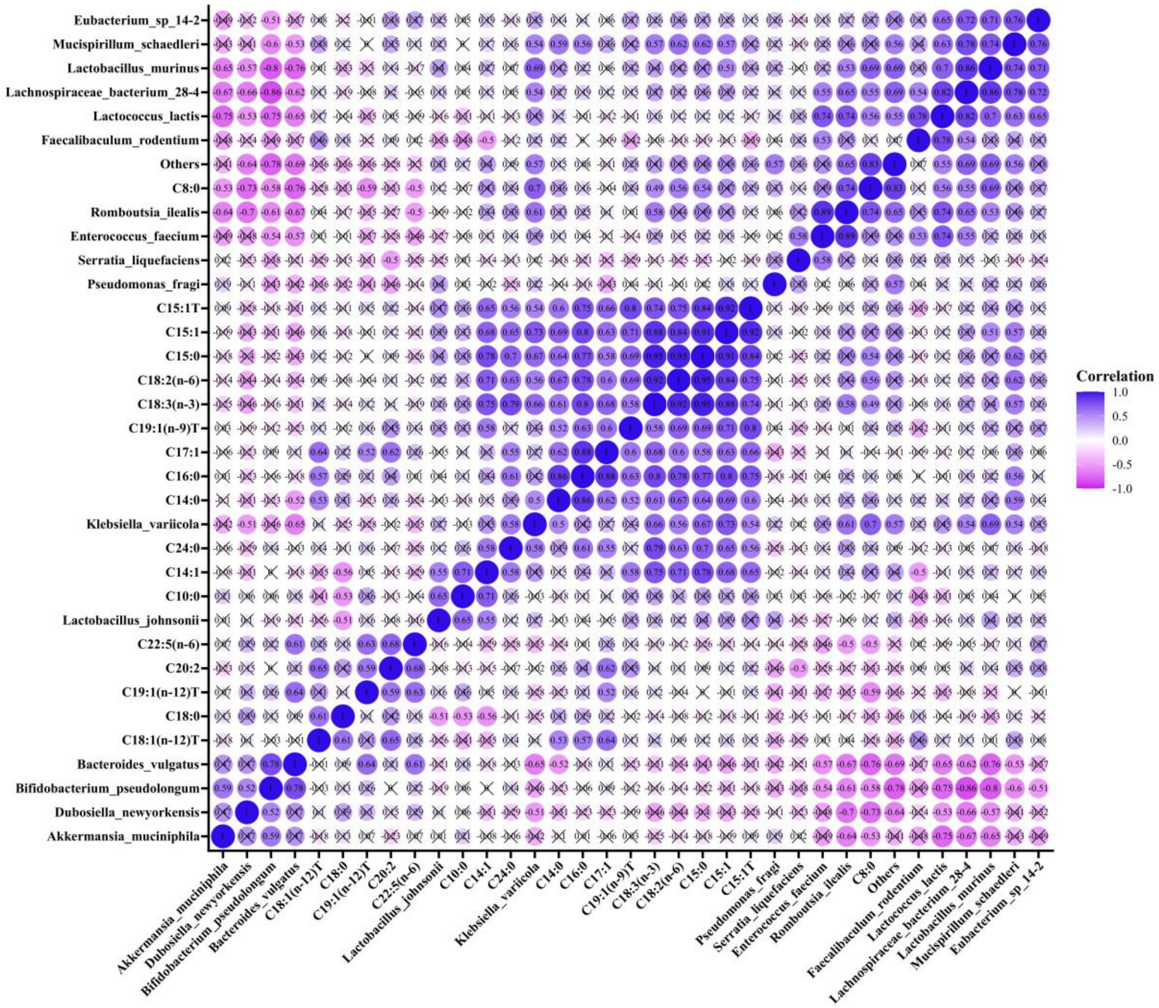

Supplementary Figure S7: Spearman's correlation analysis between species of gut bacteria and hepatic fatty acids.
